# Identification of the Medicine‐Related Priorities for People With Parkinson’s During Hospitalisation: A Systematic Review Using Meta‐Ethnography

**DOI:** 10.1155/padi/8428752

**Published:** 2026-08-25

**Authors:** Anna Delf, David Wright

**Affiliations:** ^1^ University of Lincoln, Lincoln, UK, lincoln.ac.uk; ^2^ School of Healthcare, University of Leicester, Leicester, UK, le.ac.uk

## Abstract

**Background:**

People with Parkinson’s (PwP) experience disproportionately higher rates of hospitalisation, where they encounter many medication‐related challenges. Although interventions have been developed to address these issues, medication‐related problems persist, and the patient’s voice is largely absent from their design.

**Objectives:**

To explore medication‐related priorities during hospitalisation from the perspective of PwP to inform the development of future patient‐led interventions.

**Methods:**

A systematic review using meta‐ethnography was conducted and registered with PROSPERO. A SPICE‐informed search (Setting, Perspective, Intervention, Comparison, Evaluation) was undertaken across MEDLINE, PsycINFO, CINAHL, Embase and Google Scholar in June 2025. Eligible qualitative and mixed‐method studies explored the perspectives of PwP regarding inpatient medication‐related care. Two reviewers independently screened studies using Covidence. First‐ and second‐order constructs were synthesised into third‐order constructs representing medication‐related priorities. Reporting followed Enhancing Meta‐Ethnography Reporting Guidance (eMERGe), and methodological quality was appraised using the Critical Appraisal Skills Programme (CASP) checklist.

**Results:**

Four studies met the inclusion criteria. Four overarching themes reflected the core medication‐related priorities of PwP during hospitalisation: timely and accurate medication administration, autonomy, access to skilled and knowledgeable staff, and advocacy to support patient needs. These priorities informed the development of an interpretive conceptual framework illustrating the interaction between PwP medication‐related priorities and hospital systems.

**Conclusions:**

Findings suggest the need to strengthen systems supporting timely medication administration, enable self‐administration where appropriate, promote flexibility and person‐centred care, and implement Parkinson’s clinical champions to advocate for and support PwP throughout hospitalisation.

## 1. Introduction

Parkinson’s is the fastest growing neurological condition globally [1], with approximately 1 in 37 people in the UK diagnosed in their lifetime [2]. Although there is currently no cure to this progressive, neurodegenerative illness, medications remain the core treatment for people with Parkinson’s (PwP) [3, 4]. Motor symptoms are primarily managed with the dopamine precursor levodopa, dopamine receptor agonists or agents that inhibit enzymatic degradation to increase dopamine availability in the central nervous system [5]. In addition, non‐motor symptoms—such as hallucinations, depression and postural hypotension—may require separate therapeutic interventions [5, 6]. Consequently, PwP often require complex medication regimens that are carefully managed and tailored to their individual needs.

PwP have a significantly higher likelihood of hospital admission compared to age‐matched controls, primarily due to complications arising from manifestations of the disease as it progresses [7]. For example, frozen gait and postural hypotension contribute to an increased incidence of falls and fractures [8], while autonomic dysfunction predisposes patients to genitourinary infections [9]. A retrospective review by Shabidat et al. [10] identified that respiratory infections were the most common cause of hospitalisation among PwP, reflecting the heightened risk of dysphagia leading to aspiration pneumonia. Once admitted, PwP typically face extended hospital stays—averaging 2–14 days longer than those without Parkinson’s [11]. It has been reported that 20% of hospitalised PwP will experience loss of motor control with 44% of patients never returning to their baseline functional status [12].

The challenges associated with inpatient pharmaceutical management of PwP are well substantiated in the literature. Delayed, inaccurate or omitted Parkinson’s doses have been shown to prolong length of hospital stay and have been significantly associated with inpatient deterioration [12–14]. In severe cases, abrupt withdrawal of dopaminergic therapy can induce a hypodopaminergic state, potentially leading to Parkinsonism–hyperpyrexia syndrome (PHS)—a rare but serious and life‐threatening complication [15].

As well as delayed and omitted doses, problematic prescribing during hospitalisation can worsen symptom control for PwP. For example, it is documented that medications contraindicated in Parkinson’s such as haloperidol and metoclopramide have been inappropriately prescribed for PwP, which can lead to symptom deterioration [16, 17]. Rotigotine patches are frequently used as an alternative to oral dopaminergic therapy for inpatients when the enteral route is unavailable. Evidence shows that incorrect conversions from oral to transdermal Parkinson’s medications can result in inappropriate dosing, leading to loss of symptom control, hallucinations and delirium in hospitalised patients [18]. Furthermore, complex medication regimens and varying availability of strengths and preparations can cause confusion among healthcare professionals, giving rise to prescribing and administration errors [19, 20].

It is internationally recognised that a shared care approach between the patient and multi‐disciplinary team should be adopted to treat Parkinson’s [21]. This requires moving from ‘physician‐based care’ to ‘collaborative patient centred care’ to ensure that the patients play an active role in their own treatment [22]. Although there is a substantial body of literature on the medical management of hospitalised PwP, far less attention has been given to the patient’s own voice and priorities regarding pharmaceutical care. Furthermore, the bulk of studies relating to patient medication priorities have been through a community lens without a hospital focus [23, 24]. While Dunsmure et al. [25] offered a valuable commentary into patient perspectives on inpatient medication‐related issues, no qualitative synthesis has yet been conducted in this area. Therefore, the overall aim of this study was to identify the pharmaceutical‐related priorities from the perspective of hospitalised PwP, with the long‐term aim of informing the future development of a complex, patient‐led intervention to improve inpatient pharmaceutical care.

## 2. Methodology

The RETREAT criteria, developed by Booth et al. [26], underpinned the selection of the qualitative evidence synthesis (QES) method for this review. RETREAT was developed to ‘inform selection of the most appropriate QES method to answer research questions addressed by qualitative research’ [26]and recommends consideration of seven domains: Review question, Epistemology, Time/Timescale, Resources, Expertise, Audience and purpose, and Type of data [26]. The selection of meta‐ethnography was informed by consideration of these domains, with the review question, epistemological orientation, intended audience and purpose, and anticipated type of data being particularly influential.

The review sought to develop an interpretive understanding of the medication‐related priorities of PwP during hospitalisation to inform the future development of a patient‐led intervention. It was underpinned by an interpretivist epistemological orientation, with the aim of developing a higher‐level, interpretive understanding of patients’ medication‐related priorities through the synthesis of qualitative accounts. The anticipated inclusion of conceptually rich qualitative studies was considered well suited to the comparative and interpretive processes of meta‐ethnography. Furthermore, the purpose of the review extended beyond describing patient experiences to generating findings that could inform future intervention development and clinical practice. Although other QES methods may also have been appropriate, meta‐ethnography was considered preferable because its emphasis on translating concepts across studies offered greater potential to develop an explanatory understanding of patients’ medication‐related priorities than a primarily descriptive synthesis.

### 2.1. Search Strategy

This systematic review protocol was published on PROSPERO (registration: CRD420251058431) on 27 May 2025, with minor updates to the search strategy and inclusion criteria reflected in later versions. This study was conducted in accordance with the eMERGe reporting criteria [27] and adhered to the Preferred Reporting Items for Systematic Reviews PRISMA guidelines [28].

Our search strategy was guided by the SPICE acronym (Setting, Perspective, Intervention, Comparison, Evaluation) [29]. Interested in qualitative outcomes, we did not include a search term for the ‘Comparison’ element. Instead of attempting to identify all possible terms to describe patient preferences, concerns and priorities regarding their medicines, we applied qualitative research methodology terms to address the ‘Evaluation’ component. Recognising that this approach produced a large number of potential hits, we intentionally adopted this strategy to minimise the likelihood of missing relevant research papers.

We combined four major concepts, with their associated search terms, using the Boolean logic terms ‘or’ and ‘and’ as displayed in Table 1. Subject headings and keywords were adapted as required to align with the requirements of each database being used. To ensure comprehensive coverage, we applied our search across four major databases—MEDLINE *(searched 03 June 2025)*, PsycINFO *(searched 04 June 2025)*, CINAHL *(searched 04 June 2025)* and Embase *(searched 05 June 2025)*. Google Scholar *(searched 05 June 2025)* was also used to identify any further relevant studies. Backward citation searching of the seed references was then carried out by manually screening the reference list titles and abstracts for any further studies that met the eligibility criteria as per the Guidance on terminology, application and reporting of citation searching: the TARCiS statement [30].

**TABLE 1:** Search terms.

SPICE	Search term *(combined with Boolean operator: ‘Or’)*	Major concept *(combined with Boolean operator: ‘And’)*
Setting	‘hospital∗’.ab,kf,ti,tw. ‘perioperative∗’.ab,kf,ti,tw. ‘surgery∗’.ab,kf,ti,tw. ‘emergency care∗’.ab,kf,ti,tw. ‘inpatient∗’.ab,kf,ti,tw. Hospitals.mp. Perioperative Period.mp. Hospitalization.mp. Patient Admission.mp. Secondary Care.mp. ‘ward∗’.ab,kf,ti,tw. ‘secondary care∗’.ab,kf,ti,tw. Inpatients.mp.	Hospitalisation

Perspective	‘Parkinson∗’.ab,kf,ti,tw. ‘paralysis agitans∗’.ab,kf,ti,tw. Parkinson Disease.mp.	Parkinson’s

Intervention	‘pharmac∗’.ab,kf,ti,tw. ‘drug∗’.ab,kf,ti,tw. ‘prescription∗’.ab,kf,ti,tw. ‘medicine∗’.ab,kf,ti,tw. ‘medication∗’.ab,kf,ti,tw. ‘drug therapy∗’.ab,kf,ti,tw. Drug Therapy.mp.	Drug Therapy

Evaluation	‘qualitative∗’.ab,kf,ti,tw. ‘focus group∗’.ab,kf,ti,tw. ‘interview∗’.ab,kf,ti,tw. ‘ethnograph∗’.ab,kf,ti,tw. ‘survey∗’.ab,kf,ti,tw. ‘questionnaire∗’.ab,kf,ti,tw. ‘mixed‐method∗’.ab,kf,ti,tw. ‘mixed method∗’.ab,kf,ti,tw. Focus Groups.mp. Qualitative Research.mp. ‘Surveys and Questionnaires’.mp.	Qualitative Research

### 2.2. Screening Process

All search results were imported into COVIDENCE software manager [31] to facilitate efficient de‐duplication and screening of literature [32]. A ‘double screening’ study selection approach was carried out by lead authors AD and DW, as recommended by Waffenschmidt et al., [33] to increase the reliability and rigour of our review. In the first stage, the title and abstracts of all retrieved articles were screened and subsequently the full texts were reviewed for inclusion. Any discrepancies were documented and resolved through a joint meeting of study authors to confirm eligibility.

### 2.3. Eligibility Criteria

As the study focus is the pharmaceutical priorities of PwP in hospital, we excluded studies set in community without a secondary care perspective. Participants were required to have a previous diagnosis of Parkinson’s and the patient’s perspective on inpatient pharmaceutical care needed to feature prominently in the findings. We sought primary qualitative or mixed‐method peer‐reviewed studies published in English with the aim of capturing the patient’s voice. No limitations were placed on the year or geographical location of publication to ensure the inclusion of all potentially relevant literature.

### 2.4. Data Extraction

Our review followed the meta‐ethnography in healthcare research guidelines by Sattar et al. [34], which built upon the original seven phases outlined by Noblit and Dwight Hare [35]. This framework was used to provide greater clarity in navigating the meta‐ethnography synthesis process for this review.

Initially, key study characteristics such as date published, author information and participant details were extracted by the primary author (AD) using an adapted version of the Cochrane data extraction form [36].

In line with meta‐ethnographic principles, we extracted all participant narratives (first‐order constructs) and the corresponding primary author interpretations (second‐order constructs) relating to the pharmaceutical care priorities of PwP. As recommended by the eMERGe criteria, we reviewed all sections of each primary study for constructs rather than only the ‘findings’ section, to ensure important conceptual data was not overlooked [27]. These constructs were then organised into adjacent columns within a Microsoft Excel spreadsheet, with an additional column reserved for interpretive notes, allowing us to track the development of emerging themes and ideas throughout the process.

We noted that the perspectives of family members or caring partners (CPs) were included in each of the selected studies. Given that up to 90% of PwP experience communication difficulties—including impairments in voice (phonation), speech and language [37]—the retrieved studies emphasised the role CPs play in communicating on behalf of the patient and advocating for their needs. Therefore, we also extracted all first‐order constructs related to CPs into a separate Excel spreadsheet to facilitate comparison between the priorities of PwP and CPs.

To enhance rigour and consistency in the data extraction process, the second reviewer, DW, independently repeated the extraction for 25% of the included studies, enabling identification of any discrepancies.

### 2.5. Translation and Synthesis

Each study was initially read and re‐read by the primary (A.D.) to develop a comprehensive understanding of its content. To explore how the studies related to one another, both reviewers (A.D. and D.W.) independently coded recurring concepts across the literature. Where first‐ and second‐order constructs related to multiple concepts, we created additional rows in the Microsoft Excel spread sheet to reflect these overlaps. These were then discussed collaboratively between both authors (A.D. and D.W.) to assess the relevance and inclusion of each identified concept. Next, we examined whether the relationships between concepts across studies were reciprocal (where findings reinforced each other) or refutational (where findings contrasted) [34]. We then conducted conceptual translation, comparing and integrating findings across studies. Through this process, we combined the identified concepts into broader, higher‐level themes, leading to the development of third‐order constructs. A line‐of‐argument (LOA) synthesis was carried out, as recommended by Sattar et al. [34], which involved integrating the third‐order constructs into an overarching interpretive explanation of how patient priorities may conflict with hospital medication‐related systems. This final stage moved beyond thematic aggregation to generate a conceptual interpretation of the pharmaceutical priorities of hospitalised PwP.

### 2.6. Risk of Bias

The Critical Appraisal Skills Programme (CASP) checklist was selected as it is widely used and validated within healthcare research [38]. The initial assessment of risk of bias was conducted by the first author (AD), with senior author (DW) independently reviewing 25% of the papers to enhance confidence in our approach.

### 2.7. Confidence Assessment

The GRADE‐CERQual approach was used to assess confidence in the findings identified through our qualitative synthesis [39]. This assessment considered the four key components of the GRADE‐CERQual framework: methodological limitations, coherence, adequacy of data and relevance, to determine the overall confidence in the study’s findings.

## 3. Results

### 3.1. Outcome of Search Process

**FIGURE 1:**
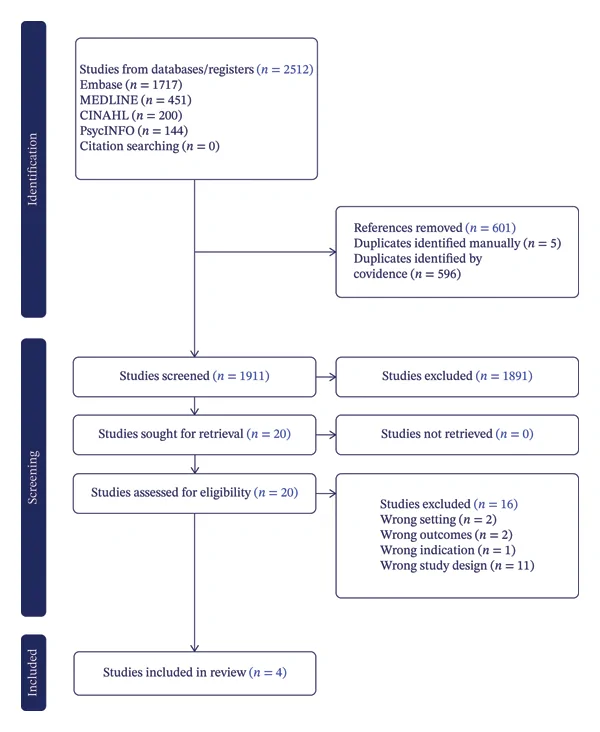
PRISMA diagram.

The search yielded 2512 potentially relevant studies across four major databases. Reference lists of selected studies and a Google Scholar search were also examined but did not identify any additional eligible studies. Following screening and selection, four papers were included for data extraction, as illustrated in the PRISMA diagram (Figure 1).

### 3.2. Study Characteristics

All studies included in our review are presented in Table 2. Each study employed a qualitative design, utilising either semi‐structured interviews or focus groups to explore participants’ perspectives. All studies identified themes related to the experiences of PwP during hospitalisation, and we have displayed the medication‐related themes specific to each in Table 2.

**TABLE 2:** Study characteristics.

ID	Title	Authors	Date	Country	Objective	Type of hospital admission	Participants	Methodology	Medication‐related themes
1	*The Perioperative Experience of Patients with Parkinson’s Disease: A Qualitative Study* [40]	Anderson, L.C.; Fagerlund, K.	Feb 2013	USA	To understand the perioperative experiences of patients with Parkinson’s using their own words	Elective surgical procedures requiring nil‐by‐mouth status for at least 6 hours prior to surgery	13 PwP	Semi‐structured face‐to‐face interviews	*Medication timing may not fit with hospital routine* *Patients know their own bodies and the best medication regimen* *Education needed for hospital sta*ff

2	*What Really Matters? A Multi-View Perspective of One Patient’s Hospital Experience* [41]	Edwards, K.J.; Duff, J.; Walker, K.	Nov 2014	Australia	To explore perceptions of a patient’s hospital experience, identifying what mattered to the patient and family, and whether healthcare providers were aware of these needs	Planned hospital admission for deep brain stimulation (DBS) surgery	1 PwP 1 CP 7 HPs	Semi‐structured face‐to‐face interviews	*Medication management*

3	*More Than Medications: A Patient-Centred Assessment of Parkinson’s Disease Care Needs During Hospitalization* [42]	Shurer, J.; Golden, S.L.S.; Mihas, P.; Browner, N.	Sep 2023	USA	To identify challenges and opportunities for patient‐centred Parkinson’s care in hospital settings	Planned or unplanned stays of ≥ 24 h (84% unplanned, 16% planned)	12 PwP 13 CPs	Focus groups using semi‐structured guide	*Numerous hurdles with PD medications management lead to dissatisfaction with hospital care among the participants*

4	*Medication Timing Errors for Parkinson’s Disease: Perspectives Held by Caregivers and People with Parkinson’s in New Zealand* [43]	Buetow, S.; Henshaw, J.; Bryant, L.; O’Sullivan, D.	Nov 2010	New Zealand	To explore lay perspectives of factors contributing to medication timing errors in hospital and community settings	Not recorded	13 PwP 7 CPs	Semi‐structured telephone interviews	*Abrupt withdrawal of PD medication* *Lack of professional knowledge and caring behaviour* *Devaluation of the lay role* *Instructions wrong, vague, or misread*

Anderson and Kate [40] focused primarily on PwP but incidentally included the voice of a patient’s wife, while Edwards et al. [41], Shurer et al. [42], and Buetow et al. [43] explicitly included the perspectives of CPs. The studies were conducted across different geographical locations: Anderson and Kate [40] and Shurer et al. [42] in the United States; Edwards et al. [41] in Australia; and Buetow et al. [43] in New Zealand.

While all studies broadly aimed to capture the perspectives of PwP during hospitalisation, Edwards et al. [41] differed in focus. Its objective was to explore the overall hospital journey and experience of a single patient with Parkinson’s syndrome, contrasting the patient’s perception with that of their CP and healthcare providers (HPs). Anderson and Kate [40] and Edwards et al. [41] captured the experiences of PwP during planned surgical admissions, offering insights into post‐operative needs such as pain management. Shurer et al. [42] explored perspectives from both planned and unplanned hospitalisations, highlighting differences between elective and emergency admissions. Buetow et al. [43] examined pharmaceutical care experiences across both community and hospital settings. For the purposes of this review, we extracted only the data—specifically, participant quotes and primary author interpretations—relevant to inpatient care, in line with our eligibility criteria.

### 3.3. Outcome of Risk of Bias Assessment

The risk of bias assessment, based on the CASP qualitative checklist, demonstrated that all the retrieved studies were deemed to be generally methodologically sound. Most studies had clearly defined aims, appropriate qualitative designs, and robust ethical considerations. Minor limitations were noted in some studies regarding the researcher–participant relationship and, in a few cases, the rigour of data analysis. Overall, the studies provide credible insights into the experiences of PwP and their CPs. The detailed outcome of the assessment is displayed in Table 3.

**TABLE 3:** CASP qualitative study checklist.

Study reference	Anderson & Fagerlund, 2013	Edwards et al., 2014	Shurer et al., 2023	Buetow et al., 2010
Was there a clear statement of the aims of the research?	Yes	Yes	Yes	Yes
Is a qualitative methodology appropriate?	Yes	Yes	Yes	Yes
Was the research design appropriate to address the aims of the research?	Yes	Yes	Yes	Yes
Was the recruitment strategy appropriate to the aims of the research?	Yes	Yes	Yes	Yes
Was the data collected in a way that addressed the research issue?	Yes	Yes	Yes	Yes
Has the relationship between researcher and participants been adequately considered?	Partially	Partially	Partially	Partially
Have ethical issues been taken into consideration?	Partially	Yes	Yes	Yes
Was the data analysis sufficiently rigorous?	Partially	Partially	Yes	Yes
Is there a clear statement of findings?	Yes	Yes	Yes	Yes
How valuable is the research?	Yes	Yes	Yes	Yes

**Outcome**				

Positive/methodologically sound	X	X	X	X
Negative/relatively poor methodology				

## 4. Overall Findings

The concepts identified across the included studies were reviewed and found to be closely aligned in focus. Consequently, a reciprocal translation approach was adopted, followed by a synthesis of the findings.

Although the perspectives of PwP and their CPs were initially extracted and analysed separately, the concepts that emerged were largely consistent across both groups and reflected shared priorities regarding inpatient medication management. Rather than representing distinct or conflicting perspectives, the accounts of CPs frequently complemented and reinforced those of PwP by describing the same medication‐related challenges from an advocacy and caregiving perspective. This is particularly relevant in Parkinson’s, where communication difficulties [37] and cognitive changes [44] may impact the ability to consistently communicate medication needs during hospitalisation. Therefore, synthesising the perspectives of PwP and CPs together enabled a richer understanding of hospital medication‐related priorities while preserving the shared meanings across participant groups. Accordingly, both first‐order participant accounts and second‐order constructs were translated across studies to develop the overarching themes presented in Supporting File 1.

From this synthesis, four themes were identified that represent the key medication‐related priorities of PwP during hospitalisation as displayed in Table 4. Notably, Paper 3 was found to be the most conceptually rich, offering a particularly detailed and comprehensive account of both patient and carer experiences with inpatient pharmaceutical care.

**TABLE 4:** Summary of meta‐ethnography results.

Patient priority	Subtheme	Study ID			
		1	2	3	4
Need for timely and accurate medication	Timing	✔	✔	✔	✔
	Availability			✔	
	Omission			✔	✔
	Inaccuracy	✔			✔

Desire for autonomy over pharmaceutical care	Patient Expert	✔		✔	✔
	Self‐Administration	✔	✔	✔	✔

Access To Skilled, Knowledgeable and Caring Staff	Knowledge	✔	✔	✔	✔
	Prioritisation		✔	✔	✔
	Communication		✔	✔	✔
	Compassionate Care		✔	✔	✔

Value of advocacy	Healthcare Staff Advocate	✔	✔	✔	
	Caring Partner Advocate	✔		✔	✔

### 4.1. Need for Timely and Accurate Medication Administration

#### 4.1.1. Timing

A recurring frustration for patients was the lack of timely and consistent medication administration, which negatively influenced their hospital experience [40–43]. Although some participants mentioned poor medication management within the Emergency Room (ER), issues with timely and accurate medication administration were not isolated to a specific point of the inpatient journey [42]. While unplanned admissions were described as ‘chaotic’ [42], even patients with planned, elective procedures still experienced delays in receiving their prescribed doses [40–42].

*‘All participants reported issues with consistent and timely delivery of Parkinson’s Disease medications at all stages of hospitalization’* [42].

Hospital staff sometimes adhered rigidly to routines, limiting individualised care. Regular administration schedules in hospitals did not always match the times that patients required doses, and, in some instances, staff had a *‘hard time understanding why’* PwP [40]. In other cases, procedures surrounding patient medication handling and prescribing led to unnecessary delays in doses [42].

#### 4.1.2. Availability

Participants were concerned about the lack of availability of regular Parkinson’s medications [42]. This led to delays in receiving doses or medication omissions. Although some patients brought in their own medications or had CPs offering to do so, there were many reports of staff being resistant to using the patient’s own supply

*‘They said the hospital did not have it (carbidopa). They said “Well we’ll have to see if pharmacy can get it.” And I offered to bring it and they did not want me to bring it. It was the third day they finally got it straightened out, so I don’t think he had carbidopa until just before time to leave’* [42], CP.

#### 4.1.3. Omission

As well as delays in timing, there were accounts of participants missing their doses completely. At times, this was unintentional due to supply issues [42]; however, there were other instances where Parkinson’s medications were intentionally, but inappropriately stopped [43].

#### 4.1.4. Inaccuracy

PwP and their CPs described instances where medications were prescribed or administered incorrectly. This was attributed to confusion over Parkinson’s medication preparations [40], or *‘instructions being vague, wrong or misread’* [43] Some participants indicated that the medication knowledge of PwP or their CPs was not leveraged by staff to gain an accurate drug history [42, 43].

According to (patient’s daughter) timing errors could have been avoided ‘*if they (hospital staff) had only asked me (the caregiver)—I had the latest script’* [43].

### 4.2. Desire for Autonomy Over Pharmaceutical Care

#### 4.2.1. Patient Expert

Many patients voiced expert understanding of their own bodies and individual pharmaceutical care needs [40, 42, 43]. They were aware of the time frame and frequency that Parkinson’s medications were required to best control their own symptoms

*‘And it’s something that long-term Parkinson’s patients have dealt with for quite a few years and is experimented with, with doses and time frames. And your body kind of tells you what works and doesn’t work’* [40], PwP.

However, patients’ expertise in managing their own care was not always recognised or utilised by hospital staff [40, 42, 43]. Rather than engaging in collaborative discussions around dosing schedules and pharmaceutical care needs, staff overlooked or failed to act upon the specific medication requirements of PwP

*‘Several participants suggested that hospital staff wanted to take control of the PD medications and did not seek or respect the insights or perspective of the person with PD or their family’* [43]

#### 4.2.2. Self‐Administration

PwP expressed a desire to maintain control of their own medication supply while in hospital [41, 42]. Some patients wished to keep their tablets close to them and were reluctant to give them to hospital staff after being admitted. Additionally, some patients expressed a preference for self‐administering their medication during hospitalisation to ensure doses were taken at the appropriate times [41, 42]

*‘I wish there was a way for patients who are self-aware to be able to be more self-dosing while they are in the hospital, with some limit per day. They often know their needs better than any staff can’* [42], PwP.

Furthermore, although not initially intended, PwP or their CPs often resorted to self‐administering medication when hospital systems failed to prescribe or deliver doses on time [42]

*‘I mentioned I had Parkinson′s disease when it was time to take my medication. I said I brought medication, but they insisted they draw it from pharmacy rather than use my medication. My medication was delayed and eventually I took my own’* [42], PwP.

### 4.3. Access to Skilled, Knowledgeable and Caring Staff

#### 4.3.1. Knowledge

PwP were frequently dissatisfied with the level of staff knowledge of Parkinson’s’ disease and its associated pharmaceutical care needs [40, 42, 43]. This lack of understanding contributed to feelings of mistrust toward HPs, while staff with specialist Parkinson’s expertise offered reassurance and increased patients’ confidence in their care [42]

*‘I’m still surprised at how many staff don’t understand Parkinson’s. You run into a wall, and they don’t know anything. And it’s been going on a long time. You’d think they’d get the word’* [40], PwP.

#### 4.3.2. Prioritisation

Instances were identified where Parkinson’s medications were not prioritised by hospital staff. For example, in Edwards et al. 2014 [41], there were reports of medication delays when the ‘ward did not appear busy’. Similarly, Buetow et al. described how hospital staff were ‘standing around’ and engaging in ‘casual talk’ although doses were not on time [43]. In another example, the priority was treating the presenting condition without considering the urgency of administering Parkinson’s tablets [42]

*‘My medications were always late but I didn’t bother addressing it. I was never there long enough to make it worthwhile to change it. And all the focus was on my urinary tract infection’* [42], PwP.

#### 4.3.3. Communication

Clear and honest communication from healthcare staff was important for PwP [40–42]. Participants reported issues with staff members not listening to them and ‘*struggling to have their views heard and valued in hospitals* [43]’. Similarly, trust deteriorated when medications *‘were substituted or re-arranged without explanation* [42]’. Conversely, staff who openly acknowledged the presence of a Parkinson’s diagnosis [42] or those who communicated specifics of care needs were at times *‘responsible for the alleviation of fear* [41]’.

#### 4.3.4. Compassionate Care

Patients described instances where staff demonstrated a lack of compassionate care, appearing to overlook their Parkinson’s diagnosis and associated needs [42, 43]. In some cases, PwP and their CPs raised concerns about medication management but felt ignored or dismissed by staff

*‘they did not like being corrected, any of them … I felt that they would have been happier if I had not been there and chased them up on times’* [43], PwP.

By contrast, patients felt more at ease and reassured when staff were perceived as compassionate [41–43]

‘*Feeling cared for was identified by the recipients as having positively impacted their experience’* [41].

### 4.4. Value of Advocacy

#### 4.4.1. Healthcare Staff Advocate

Individual members of staff advocated for the needs of patients with Parkinson’s and therefore could reassure and improve inpatient experience for PwP [40–43]

*‘I found one nurse who was willing to fight for me. She was great. She was really making a point of making sure I got my meds, and leaving notes for the next shift’* [40], PwP.

#### 4.4.2. Caring Partner Advocate

CPs played a supportive role during the inpatient journey of patients with Parkinson’s. As well as providing information on medication histories [42, 43], they challenged and reduced prescribing or administration errors for their loved ones. CPs would also step in where the hospital system had failed and bring in medication supply or administer doses for the patient

*‘…and so a couple times I gave medication to him when he needed to have it and then I just told them that I’d already given it to him. I know they were not happy but we could not wait. It was very hard to get through that this is time sensitive*’ [42], PwP.

### 4.5. Conceptual Model

**FIGURE 2:**
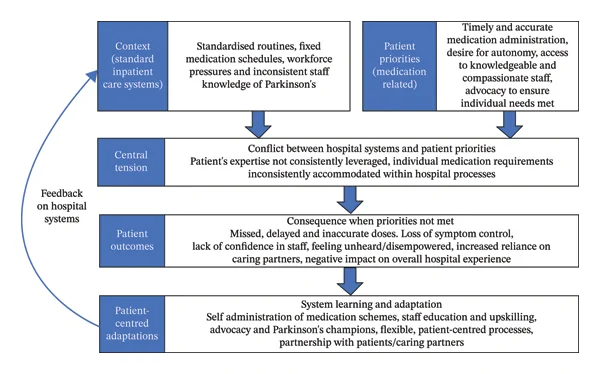
Conceptual framework: An interpretive framework of the tension between hospital systems and medication‐related priorities of people with Parkinson’s.

Following the identification of the four overarching themes, an LOA synthesis was undertaken to explore how these concepts related to one another and contributed to the wider understanding of pharmaceutical care priorities for hospitalised PwP. The resulting conceptual framework (Figure 2) integrates the relationships between hospital systems, patient priorities, the consequences when these priorities are not met, and factors that may help to reduce this tension. The framework represents an interpretation of how the identified concepts interact across studies, moving beyond individual themes to provide an overarching explanation of the inpatient pharmaceutical care experiences of PwP.

### 4.6. GRADE‐CERQual Results

Overall, the GRADE‐CERQual assessment indicated that most findings were of high confidence. The four overarching priorities identified by PwP were all rated high and were all supported by rich, consistent data from all four studies, highlighting their central relevance to the research question. Some subthemes such as communication and compassionate care were supported by fewer studies and variable examples, leading to moderate or low confidence ratings. The subthemes of medication availability were rated low due to inadequacy of data, but this finding was implied across the literature and had thematic links to the overarching patient priority of ‘need for timely and accurate medication administration’. A detailed breakdown of results is provided in Supporting File 2.

## 5. Discussion

This meta‐ethnography moved beyond aggregating patient experiences by translating concepts across studies to generate an interpretive understanding of pharmaceutical care priorities during hospitalisation. Although based on only four conceptually rich studies, the synthesis identified four interconnected priorities—timely and accurate medication administration, autonomy, knowledgeable and compassionate staff, and advocacy—that appear to represent responses to a common challenge: the tension between standardised hospital systems and the individualised medication needs of PwP. While these findings should be interpreted cautiously, the application of GRADE‐CERQual strengthens confidence in the higher‐order themes and supports their use as hypothesis‐generating concepts for future research and service development.

It is well known that medication timing errors occur for PwP in hospitals and care homes [20, 45, 46]. Our study suggests that, from the patient perspective, improving medication timing, accuracy and availability would enhance inpatient experience for PwP. As per the NICE (2018) Parkinson’s Disease Quality Standards [47], hospital inpatients should receive their dose of levodopa within 30 min of their usual administration time. However, despite well‐publicised initiatives such as the Leeds QI Project [48] and the Parkinson’s UK ‘Get it on Time’ campaign [49], the last Parkinson’s UK Audit 2025 report [45] demonstrated that only one in eight of audited inpatients received every dose on time, highlighting the ongoing need for improvement. These findings suggest that medication delays are not merely isolated errors in practice but may reflect a broader tension between standardised hospital processes and the highly individualised medication regimens required by PwP.

Autonomy over pharmaceutical care was consistently identified as a priority during hospitalisation and was supported by a high‐confidence CERQual finding. Previous research has shown that empowering PwP to participate in shared decision‐making and take greater ownership of their care can improve outcomes [50, 51]. Parkinson’s UK has produced guidance on developing self‐administration (SAM) policies for hospital staff to ensure that PwP can safely manage their medications during hospitalisation [52]. However, although a lack of data, the scale of uptake of SAM in hospitals has been reported to be poor, possibly due to the increased staff workload associated with the scheme [53–55]. Our findings suggest that expanding opportunities for eligible PwP to self‐administer medication during hospitalisation could lead to improved overall patient experiences.

Access to knowledgeable, skilled and compassionate healthcare professionals was supported by a high‐confidence CERQual finding, indicating that participants valued staff who understood Parkinson’s, prioritised timely medication administration and communicated effectively. This aligns with a recent qualitative study by Kinger et al. [56] in which PwP emphasised the importance of being listened to by healthcare staff, acknowledged and treated as individuals. Multiple schemes have been implemented to improve Parkinson’s education such as physical reminders such as posters and stickers [57], online courses and multimodal training programmes [48, 57]. To support timely specialist involvement, inpatient alert systems have also been used to inform specialists of admissions concerning PwP [58]. Despite this, access to specialist expertise remains inconsistent [59], highlighting the continued need to upskill and educate the workforce as laid out in the Parkinson’s UK 2025 audit report [45].

The value of advocacy was consistently demonstrated throughout the studies included in the review and was supported by a high‐confidence CERQual finding. Patients benefited from proactive, individual members of staff or CPs providing personalised support. At times, CPs had to intervene where the hospital system had failed to meet the specific needs of PwP. This finding aligns with other studies highlighting the crucial role CPs play in supporting PwP [59, 60].

Bloem et al. proposed a network model of person‐centred Parkinson’s care that emphasises identifying individual patient priorities rather than applying standardised approaches [61]. Consistent with this perspective, our conceptual framework (Figure 2) suggests that many medication‐related problems during hospitalisation arise from a mismatch between standardised hospital systems and the individualised medication needs of PwP. These findings highlight the importance of flexible, person‐centred hospital care that can respond to the individual needs and priorities of PwP.

## 6. Limitations

This review has several limitations that should be considered when interpreting the findings. First, the included studies were conducted in a limited number of countries, and differences in healthcare systems, organisational structures, and medication management practices may limit the transferability of the findings. Although most review findings were assigned moderate or high confidence using GRADE‐CERQual, the subtheme related to medication availability was rated as low confidence because of limited supporting data and should therefore be interpreted cautiously.

A further limitation is the small evidence base. Of the 2512 records screened, only four studies met the inclusion criteria. This reflects the limited qualitative literature, specifically exploring medication‐related experiences of PwP disease during hospitalisation. Many qualitative studies focus more broadly on the inpatient experience, with medication management discussed only incidentally, and therefore did not meet the predefined eligibility criteria. While this increased the specificity of the review, it inevitably reduced the number of eligible studies and may have excluded broader contextual insights relevant to hospital care.

Although meta‐ethnography is often undertaken using a larger body of literature, the included studies were conceptually rich and provided sufficient first‐ and second‐order constructs relating to medication experiences. Alternative approaches, such as thematic or narrative synthesis, may have provided a descriptive summary of patient experiences but would have been less suited to exploring the relationships between themes across studies and generating a conceptual framework (Figure 2) [34].

Only one of the included studies was published within the past decade, reflecting the paucity of qualitative research that explicitly centres the medication‐related experiences of PwP disease during hospitalisation. Although inpatient practices have evolved over time, the included studies consistently described similar challenges relating to medication timing, autonomy, communication and advocacy, suggesting that these issues remain relevant.

Overall, the recommendations arising from this review should be considered as theory‐informed and hypothesis‐generating rather than prescriptive. The conceptual framework suggests that medication‐related priorities are shaped by a fundamental tension between standardised inpatient systems and the highly individualised medication needs of PwP. However, additional qualitative research across diverse healthcare settings is required to strengthen and refine these conclusions.

## 7. Conclusion and Recommendations

Our meta‐ethnography has centred the patient voice in exploring pharmaceutical care priorities during hospitalisation. The findings suggest that hospitals could consider simplified self‐administration of medication (SAM) schemes that are easy to implement for eligible PwP at the point of admission and maintained, where appropriate, throughout their inpatient stay. This approach may enhance patient autonomy and potentially reduce missed, delayed or incorrect doses. PwP should also be supported in using their own medications—at least initially—until hospital‐supplied alternatives are available. Such practices may also encourage a more compassionate and person‐centred environment within often rigid hospital systems.

The identification of Parkinson’s champions on wards could further support the effective implementation of SAM schemes, provide advocacy for PwP and promote the education and up skilling of colleagues. While this role may be suited to nurses—particularly those on care of the older person and neurology wards—it may also be appropriate for pharmacists, given their regular involvement in SAM processes. Pharmacists additionally bring pharmacological expertise, enabling them to review and potentially optimise therapy, ensuring appropriate medication management and minimising the likelihood of medication errors throughout the hospital stay.

## Funding

This work was funded by the National Institute of Health Research (NIHR) East Midlands Integrated Clinical Academic (ICA) Internship Programme.

## Ethics Statement

The authors have nothing to report.

## Conflicts of Interest

The authors declare no conflicts of interest.

## Supporting Information

Additional supporting information can be found online in the Supporting Information section.

## Supporting information


**Supporting Information 1** Supporting File 1: Presentation of first‐, second‐, and third‐order constructs. Presents examples of direct participant quotations from the included studies, alongside the associated primary authors’ interpretations. Third‐order constructs represent higher‐level analytical themes, illustrating overarching patient priorities and their associated subthemes.

**Supporting Information 2** Supporting File 2: GRADE‐CERQual confidence assessment. Presents the complete results of the GRADE‐CERQual confidence assessment for all identified patient priorities.

**Supporting Information 3** Supporting File 3: PRISMA Checklist. Presents the completed PRISMA (2020) checklist, which demonstrates adherence to systematic review requirements.

## Data Availability

The data that support the findings of this study are available in the supporting information of this article.
