# Supplementary material for: Identification of the Medicine‐Related Priorities for People With Parkinson’s During Hospitalisation: A Systematic Review Using Meta‐Ethnography
Source: Parkinsons Dis. 2026 Aug 25;2026:8428752. doi: 10.1155/padi/8428752 (PMC13504675; doi:10.1155/padi/8428752)
Supplement: Supplementary file 1 [file PADI-2026-8428752-s001.docx]

| **Section and Topic** | **Item #** | **Checklist item** | **Location where item is reported** |
| --- | --- | --- | --- |
| **TITLE** | | | Page Number |
| Title | 1 | Identify the report as a systematic review. | 1 |
| **ABSTRACT** | | |  |
| Abstract | 2 | See the PRISMA 2020 for Abstracts checklist. | 2,3 |
| **INTRODUCTION** | | |  |
| Rationale | 3 | Describe the rationale for the review in the context of existing knowledge. | 4,5 |
| Objectives | 4 | Provide an explicit statement of the objective(s) or question(s) the review addresses. | 5 |
| **METHODS** | | |  |
| Eligibility criteria | 5 | Specify the inclusion and exclusion criteria for the review and how studies were grouped for the syntheses. | 8 |
| Information sources | 6 | Specify all databases, registers, websites, organisations, reference lists and other sources searched or consulted to identify studies. Specify the date when each source was last searched or consulted. | 6 |
| Search strategy | 7 | Present the full search strategies for all databases, registers and websites, including any filters and limits used. | 6,7,8 |
| Selection process | 8 | Specify the methods used to decide whether a study met the inclusion criteria of the review, including how many reviewers screened each record and each report retrieved, whether they worked independently, and if applicable, details of automation tools used in the process. | 8 |
| Data collection process | 9 | Specify the methods used to collect data from reports, including how many reviewers collected data from each report, whether they worked independently, any processes for obtaining or confirming data from study investigators, and if applicable, details of automation tools used in the process. | 8,9 |
| Data items | 10a | List and define all outcomes for which data were sought. Specify whether all results that were compatible with each outcome domain in each study were sought (e.g. for all measures, time points, analyses), and if not, the methods used to decide which results to collect. | Not applicable to qualitative evidence synthesis; first-order constructs (participant quotes) and second-order constructs (primary author interpretations) relating to medication related priorities were extracted rather than quantitative outcomes. |
|  | 10b | List and define all other variables for which data were sought (e.g. participant and intervention characteristics, funding sources). Describe any assumptions made about any missing or unclear information. | 8 (as per eligibility criteria)  No assumptions were made regarding missing or unclear information; only data explicitly reported by the original study authors, including participant quotations, were extracted and synthesised. |
| Study risk of bias assessment | 11 | Specify the methods used to assess risk of bias in the included studies, including details of the tool(s) used, how many reviewers assessed each study and whether they worked independently, and if applicable, details of automation tools used in the process. | 10 |
| Effect measures | 12 | Specify for each outcome the effect measure(s) (e.g. risk ratio, mean difference) used in the synthesis or presentation of results. | Not applicable. Findings were synthesised through reciprocal translation via meta-ethnography approach rather than quantitative synthesis |
| Synthesis methods | 13a | Describe the processes used to decide which studies were eligible for each synthesis (e.g. tabulating the study intervention characteristics and comparing against the planned groups for each synthesis (item #5)). | Studies were screened against the predefined eligibility criteria. All eligible qualitative studies were included in a single meta-ethnographic synthesis; therefore, no additional process was required to determine eligibility for separate syntheses. |
|  | 13b | Describe any methods required to prepare the data for presentation or synthesis, such as handling of missing summary statistics, or data conversions. | First- and second-order constructs were extracted from all sections of each included study and organised in a Microsoft Excel spreadsheet alongside interpretive notes to facilitate conceptual translation and synthesis. No data conversions were required. |
|  | 13c | Describe any methods used to tabulate or visually display results of individual studies and syntheses. | Study characteristics were tabulated to summarise the included studies (Page 13). The synthesis findings were presented narratively, supported by tables illustrating the development of first-, second-, and third-order constructs (available in Supplementary File 1) |
|  | 13d | Describe any methods used to synthesize results and provide a rationale for the choice(s). If meta-analysis was performed, describe the model(s), method(s) to identify the presence and extent of statistical heterogeneity, and software package(s) used. | 9,10 |
|  | 13e | Describe any methods used to explore possible causes of heterogeneity among study results (e.g. subgroup analysis, meta-regression). | Not applicable. Statistical heterogeneity was not assessed because this was a qualitative meta-ethnography |
|  | 13f | Describe any sensitivity analyses conducted to assess robustness of the synthesized results. | Not applicable. No sensitivity analyses conducted because this was a qualitative meta-ethnography |
| Reporting bias assessment | 14 | Describe any methods used to assess risk of bias due to missing results in a synthesis (arising from reporting biases). | Not applicable. Formal assessment of reporting bias arising from missing results was not undertaken because this review synthesised qualitative evidence rather than quantitative outcome data. |
| Certainty assessment | 15 | Describe any methods used to assess certainty (or confidence) in the body of evidence for an outcome. | 10 |
| **RESULTS** | | |  |
| Study selection | 16a | Describe the results of the search and selection process, from the number of records identified in the search to the number of studies included in the review, ideally using a flow diagram. | 10,11 |
|  | 16b | Cite studies that might appear to meet the inclusion criteria, but which were excluded, and explain why they were excluded. | Not applicable |
| Study characteristics | 17 | Cite each included study and present its characteristics. | 13 |
| Risk of bias in studies | 18 | Present assessments of risk of bias for each included study. | 15 |
| Results of individual studies | 19 | For all outcomes, present, for each study: (a) summary statistics for each group (where appropriate) and (b) an effect estimate and its precision (e.g. confidence/credible interval), ideally using structured tables or plots. | Not applicable**.** This is a qualitative meta-ethnography; therefore, no summary statistics, effect estimates, or confidence intervals were calculated. Instead, findings from each included study were extracted as qualitative data (first-order participant quotes and second-order author interpretations) onto an Excel spreadsheet. These were then synthesised thematically into third-order constructs. |
| Results of syntheses | 20a | For each synthesis, briefly summarise the characteristics and risk of bias among contributing studies. | For each synthesis, we summarised the characteristics of contributing studies (page 11) and appraised methodological quality using a qualitative critical appraisal tool (page 15). |
|  | 20b | Present results of all statistical syntheses conducted. If meta-analysis was done, present for each the summary estimate and its precision (e.g. confidence/credible interval) and measures of statistical heterogeneity. If comparing groups, describe the direction of the effect. | Not applicable. No statistical syntheses or meta-analyses were conducted, as this review employed a qualitative meta-ethnographic approach. |
|  | 20c | Present results of all investigations of possible causes of heterogeneity among study results. | Not applicable in a statistical sense**.** Variation across studies was explored narratively, focusing on differences such as setting, participant groups, admission type, and study focus. (pages 11,12) |
|  | 20d | Present results of all sensitivity analyses conducted to assess the robustness of the synthesized results. | Not applicable**.** No statistical sensitivity analyses were conducted, as this is a qualitative meta-ethnography. |
| Reporting biases | 21 | Present assessments of risk of bias due to missing results (arising from reporting biases) for each synthesis assessed. | 15 |
| Certainty of evidence | 22 | Present assessments of certainty (or confidence) in the body of evidence for each outcome assessed. | Supplementary File 2 |
| **DISCUSSION** | | |  |
| Discussion | 23a | Provide a general interpretation of the results in the context of other evidence. | 23,24,25 |
|  | 23b | Discuss any limitations of the evidence included in the review. | 25, 26 |
|  | 23c | Discuss any limitations of the review processes used. | 25, 26 |
|  | 23d | Discuss implications of the results for practice, policy, and future research. | 26.27 |
| **OTHER INFORMATION** | | |  |
| Registration and protocol | 24a | Provide registration information for the review, including register name and registration number, or state that the review was not registered. | PROSPERO: CRD420251058431 (See page 6) |
|  | 24b | Indicate where the review protocol can be accessed, or state that a protocol was not prepared. | PROSPERO: CRD420251058431 https://www.crd.york.ac.uk/prospero/ |
|  | 24c | Describe and explain any amendments to information provided at registration or in the protocol. | See previous version on PROSPERO which allows comparison with current protocol https://www.crd.york.ac.uk/prospero/ |
| Support | 25 | Describe sources of financial or non-financial support for the review, and the role of the funders or sponsors in the review. | 1 |
| Competing interests | 26 | Declare any competing interests of review authors. | 1 |
| Availability of data, code and other materials | 27 | Report which of the following are publicly available and where they can be found: template data collection forms; data extracted from included studies; data used for all analyses; analytic code; any other materials used in the review. | Supplementary File 1 (Presentation of First, Second and Third Order Constructs)  Extracted data (Excel spreadsheets) are available from the corresponding author upon reasonable request. |

*From:*  Page MJ, McKenzie JE, Bossuyt PM, Boutron I, Hoffmann TC, Mulrow CD, et al. The PRISMA 2020 statement: an updated guideline for reporting systematic reviews. BMJ 2021;372:n71. doi: 10.1136/bmj.n71. This work is licensed under CC BY 4.0. To view a copy of this license, visit <https://creativecommons.org/licenses/by/4.0/>
