# Supplementary material for: Identification of the Medicine‐Related Priorities for People With Parkinson’s During Hospitalisation: A Systematic Review Using Meta‐Ethnography
Source: Parkinsons Dis. 2026 Aug 25;2026:8428752. doi: 10.1155/padi/8428752 (PMC13504675; doi:10.1155/padi/8428752)
Supplement: Supplementary file 2 [file PADI-2026-8428752-s002.docx]

**Supplementary File 1: Presentation of First, Second and Third Order Constructs**

| Direct Participant Quote Examples (First Order Constructs) | Primary Author Interpretation (Second Order Constructs) | Sub Theme | Overarching Patient Priority (Third order constructs) |
| --- | --- | --- | --- |
| “OK, so I refused the pill one time, and then an hour later needed it, and the nurses had a hard time understanding why.” PwP (1) | Medication timing may not fit with hospital routine (1) | **Timing** | **Need for Timely and Accurate Medication Administration** |
| "Sometimes the medication might be a couple of minutes either side, but I’m fairly anal about that. I like my medication when it’s on time." PwP (2) | Medication timing and administration were repeatedly mentioned... as having impacted their hospital experience (2) |  |  |
| "The medication (timing) was the issue. It wasn’t available in the pharmacy. So then of course now that starts throwing me off on timing wise.." PwP (3) | All participants reported issues with consistent and timely delivery of PD medications at all stages of hospitalization, from admission to discharge (3) |  |  |
| “They said the hospital did not have it (carbidopa). The said "Well we'll have to see if pharmacy can get it." And I offered to bring it and they did not want me to bring it.” CP (3) | Inconsistent availability of Parkinson’s medications (3) | **Availability** |  |
| “I said I brought medication, but they insisted they draw it from pharmacy rather than use my medication. My medication was delayed and eventually I took my own" PwP (3) | PwPs and CPs were concerned about the availability of PD medications and, as a result, packed and brought their medications to the hospital (3) |  |  |
| Other participants, such as P11, spoke of medication “omissions for several days” in hospital, even though it is “imperative that none of the Parkinson’s medications be halted.” CP (4) | Abrupt Withdrawal of PD Medications (4) | **Omission** |  |
| “And I said, “Excuse me, what are you giving him?” “Well, Miralax for constipation.” And I said, “Well no, that’s not—he doesn’t have constipation. He has Parkinson’s, and it better be Mirapex.” And she kinda said, “Oh no, no, no, no.” And I just said, “OK, please go check. What are you giving him?” And she came back and said, “You’re right. It’s Mirapex.”CP (1) | Spouses brought medications from home, showed nursing staff the medications their loved ones were  taking, alerted nursing staff to patients’ needs for medication, and watched to make sure that the medications given were correct (1) | **Inaccuracy** |  |
| “The charting would change (for her husband with Parkinson’s) ... they would have 8.00, 8.30 ...and I would say, “He is supposed to get his pergolide on a full stomach.” “Oh, no, no, it’s charted for ...” CP (4) | Instructions Wrong, Vague or Misread (4) |  |  |
| "The time frame I gave you for the medications is what has worked. So for you (the nurses) to arbitrarily put in a different time frame is not what my body is accustomed to. It doesn’t just adapt like that." PwP (1) | Patients know their own bodies and the best medication regimen (1) | **Patient Expert** | **Desire for Autonomy over Pharmaceutical Care** |
| “... if you are taking them everyday, you know when you need to take them; your body tells you” so ... “it is not really a sort of arranging it at the same time every day” PwP (4) | Several participants suggested that hospital staff wanted to take control of the PD medications and did not seek or respect the insights or perspective of the person with PD or their family (4) |  |  |
| "I just accepted they had to take it off us. You can’t have people in there taking their own medication when they want can you? I probably would have liked to look after my own medications, cause I’m used to that." PwP (2) | Handing over medications to the hospital staff to administer also impacted experience for the recipients (2) | **Self-Administration** |  |
| “But they were a little hesitant and, at one point, they said, “Well, why do not you give us the medication and we’ll give it to you?” And I said, “No…” I said, “I wanna keep the medication in my nightstand here so I do not get it mixed up with anything else,” and so they finally said that was fine.” PwP (3) | To ensure the correct medications were taken on time and as prescribed, many PwPs and CPs chose to administer their own medications during hospitalization (3) |  |  |
| “I wish there was a way for patients who are self-aware to be able to be more self-dosing while they are in the hospital, with some limit per day. They often know their needs better than any staff can.” PwP (3) | Many participants expressed a desire to see protocols around medication self-administration in the hospital (3) |  |  |
| “I’m still surprised at how many staff don’t understand Parkinson’s. You run into a wall, and they don’t know anything. And it’s been going on a long time. You’d think they’d get the word.” PwP (1) | Education needed for hospital staff (1) | **Knowledge** | **Access to Skilled and Knowledgeable and Caring Staff** |
| “In ER, I had to describe exactly what Parkinson's is because they'll look at my hand shaking..they just kind of look…I kind of had to explain to them and then sometimes they would ask more questions about what exactly the disease is” PwP (3) | Specific needs affecting the experience from admission to discharge were identified, including knowledge of PD medications, proper medication management (3) |  |  |
| "I do not think there was awareness of how important timing is of the Parkinson’s pills." PwP (3) | Participants reported needing to have 'continuous discussions' about their medication regimens with their care team (3) |  |  |
| “(staff) do not always understand the way the (Parkinson’s) medications work” PwP (4) | Lack of Both Professional Knowledge and Caring Behaviour (4) |  |  |
| "It didn’t make sense why (meds were late)?" PwP (2) | Both recipients expressed difficulties understanding why medications were late when the ward did not appear busy (2) | **Prioritisation** |  |
| “My medications were always late but I didn't bother addressing it. I was never there long enough to make it worthwhile to change it. And all the focus was on my urinary tract infection” PwP (3) | PwPs and CPs from multiple focus groups commented on delayed access to PD medications in the ER (3) |  |  |
| “Part of the problem,” suggested P8, “is they are short staffed ... they are rushing round all over the place ... and medication times fall by the way side.” PwP (4) | Lack of Both Professional Knowledge and Caring Behaviour (4) |  |  |
| “They were giving me different looking medications that they assured me was the same thing, just a different manufacturer or whatever it was. Every time they gave me something and I looked at it and questioned it. … it was very disconcerting for me.” PwP (3) | medications were substituted or re-arranged without explanation, which created mistrust toward HCP (3) | **Communication** |  |
| "I think he was happy (Doctor 1) had told him personally what was going on and not just fobbed him off … he has been comfortable from day one with (Doctor 1) and that’s huge … because if he is happy, I have got to be happy." CP (2) | Feeling safe and secure was highlighted throughout the recipient data as an important aspect of their experience (2) | **Compassionate Care** |  |
| “They are good at talking on, rather than listening.” (4) | Some participants reported struggling to have their views heard and valued in hospitals (4) |  |  |
| "The doctors cared too … (Surgeon) has been in every day, Doctor 2 has been in a couple of times … they have all been good."PwP (2) | “Feeling cared for was identified by the recipients as having positively impacted their experience with physical presence an indicator of caring for the patient (2) |  |  |
| “I found one nurse who was willing to fight for me. She was great. She was really making a point of making sure I got my meds, and leaving notes for the next shift.” PwP (1) | The actions of nurses could affect the perioperative experience for better or for worse (1) | **Healthcare staff advocate** | **Value of Advocacy** |
| "The doctor convinced me that they knew what they are doing with Parkinson’s as well. So that was reassuring, even though most of the focus was on the hip. I did not have to tell anaesthesiologist (about Parkinson’s diagnosis) or any of them. They were all aware. And as a matter of fact, he said to me, “Do not worry about it. We’ve got that under control and we are taking care of you.” So he was great." PwP (3) | Trust …was immediately gained when the team openly acknowledged the patient’s diagnosis of PD and demonstrated knowledge about specific considerations during hospital stays (3) |  |  |
| “I was prepared. Number one, I wasn’t alone. My daughter went with me. We had my list of medications and when I take them and how much I take. And even though they told me not to bring them, I brought my medications with me just in case I panicked and did not have them.” PwP (3) | PwP chose the date of their planned hospitalization to ensure the presence of CP during the hospital stay and after the discharge (3) | **Caring Partner Advocate** |  |
| “Now, what happened was that they did not have something I needed—I wanna say some ropinirole—they did not have it in stock. So that was a problem. And I quite honestly had my son and my husband go home and they sort of snuck it in…” PwP (3) | To ensure the correct medications were taken on time and as prescribed, many PwPs and CPs chose to administer their own medications during hospitalization. This was accomplished with or without nursing staff awareness (3) |  |  |
| According to ..(patient's daughter) timing errors could have been avoided “if they (hospital staff) had only asked me (the caregiver)—I had the latest script” CP (4) | Devaluation of Lay Role (4) |  |  |
