# Supplementary material for: Identification of the Medicine‐Related Priorities for People With Parkinson’s During Hospitalisation: A Systematic Review Using Meta‐Ethnography
Source: Parkinsons Dis. 2026 Aug 25;2026:8428752. doi: 10.1155/padi/8428752 (PMC13504675; doi:10.1155/padi/8428752)
Supplement: Supplementary file 3 [file PADI-2026-8428752-s003.docx]

## **Supplementary File 2: GRADE-CERQual Confidence Assessment**

| **Theme or Subtheme** | **Methodological Limitations** | **Coherence** | | **Adequacy of Data (study IDs of supporting studies)** | **Relevance** | **Overall Confidence** | **Explanation** |
| --- | --- | --- | --- | --- | --- | --- | --- |
| **Need for Timely and Accurate Medication** | No concerns | | High | High (1,2,3,4) | High | **High** | Finding strongly supported in evidence and directly relevant to research question. Rich and consistent amount of data containing participant quotes from PwP and CP in different settings. |
| Timing | No concerns | | High | High (1,2,3,4) | High | **High** | Strong and consistent evidence available regarding the need for timely medication in all studies. Critical priority identified by participants and no conflicting accounts. |
| Availability | No concerns | | Moderate | Low (3) | Moderate | **Low** | While relevant to research question as clearly related to medication concerns, this sub theme was only obtained from one primary study with thematic links in others. This study was set in the US so limited application to other healthcare environments. |
| Omission | No concerns | | Moderate, | Low (3,4) | High | **Moderate** | While directly relevant there are inconsistent examples such as unintentional omissions vs. intentional withdrawals. Sparse data from two studies supporting this. |
| Inaccuracy | No concerns | | Moderate | Low (1,4) | High | **Moderate** | Inaccuracy was clearly a concern but based on not especially rich data; findings are still relevant |
| **Desire for Autonomy over Pharmaceutical Care** | No concerns | | High | High (1,2,3,4) | High | **High** | Strongly supported across all studies with good richness of data. Theme of “desire for autonomy” consistent and directly relevant to the research question |
| Patient Expert | No concerns | | High | Moderate (1,3,4) | High | **High** | Well-supported in three studies in different settings with rich data. PwP expressed strong identification with being an expert in their own care. |
| Self-Administration | No concerns | | High | High (1,2,3,4) | High | **High** | Supported by all research studies with many different participants consistently expressing this medication related priority |
| **Access To Skilled, Knowledgeable and Caring Staff** | No concerns | | Consistently supported across all studies | High (1,2,3,4) | High | **High** | All studies and settings contributed to this overarching patient priority. Directly relevant to overall question and consistent accounts supporting this finding. |
| Knowledge | No concerns | | High | Moderate (1,3,4) | High | **High** | Clear examples of impact of staff knowledge on care quality. Repeated concern in all relevant data. |
| Prioritisation | No concerns | | Moderate, | Moderate (2,3,4) | High | **High** | Some varied examples, but overall supported across three studies. Good depth of data and relevant to question |
| Communication | No concerns | | Moderate | Moderate (2,3,4) | Moderate | **Moderate** | Communication was raised frequently, but with some variation and ambiguity in examples supporting this. Less direct relevance to overall research question |
| Compassionate Care | No concerns | | Moderate | Moderate (2,3,4) | Moderate | **Moderate** | Compassion was valued but inconsistently defined and less central to the main review question. Its connection to pharmaceutical care varied, and some definitions lacked clarity. |
| **Value of Advocacy** | No concerns | | High | High (1,2,3,4) | High | **High** | Theme well supported by all studies with good richness of data. Relevant to overall research question. |
| Healthcare Staff Advocate | No concerns | | High | High (1,2,3) | High | **High** | Participants valued advocacy from staff; consistently observed with strong supporting data. Directly aligns with the research question |
| Caring Partner Advocate | No concerns | | High | Moderate (1,3,4) | High | **High** | Rich commentary from PwP and CP supporting this finding, implied across all the literature, supports overall research question |
